# Motor Unit Activity during Fatiguing Isometric Muscle Contraction in Hemispheric Stroke Survivors

**DOI:** 10.3389/fnhum.2017.00569

**Published:** 2017-11-24

**Authors:** Lara McManus, Xiaogang Hu, William Z. Rymer, Nina L. Suresh, Madeleine M. Lowery

**Affiliations:** ^1^Neuromuscular Systems Lab, School of Electrical and Electronic Engineering, University College Dublin, Belfield, Ireland; ^2^Joint Department of Biomedical Engineering, University of North Carolina at Chapel Hill and North Carolina State University, Chapel Hill, NC, United States; ^3^Shirley Ryan AbilityLab, Chicago, IL, United States; ^4^Department of Physical Medicine and Rehabilitation, Northwestern University, Chicago, IL, United States

**Keywords:** motor unit, stroke, isometric fatigue, surface EMG decomposition, motor unit action potential, motor unit firing rate

## Abstract

Enhanced muscle weakness is commonly experienced following stroke and may be accompanied by increased susceptibility to fatigue. To examine the contributions of central and peripheral factors to isometric muscle fatigue in stroke survivors, this study investigates changes in motor unit (MU) mean firing rate, and action potential duration during, and directly following, a sustained submaximal fatiguing contraction at 30% maximum voluntary contraction (MVC). A series of short contractions of the first dorsal interosseous muscle were performed pre- and post-fatigue at 20% MVC, and again following a 10-min recovery period, by 12 chronic stroke survivors. Individual MU firing times were extracted using surface EMG decomposition and used to obtain the spike-triggered average MU action potential waveforms. During the sustained fatiguing contraction, the mean rate of change in firing rate across all detected MUs was greater on the affected side (-0.02 ± 0.03 Hz/s) than on the less-affected side (-0.004 ± 0.003 Hz/s, *p* = 0.045). The change in firing rate immediately post-fatigue was also greater on the affected side than less-affected side (-13.5 ± 20 and 0.1 ± 19%, *p* = 0.04). Mean MU firing rates increased following the recovery period on the less-affected side when compared to the affected side (19.3 ± 17 and 0.5 ± 20%, respectively, *p* = 0.03). MU action potential duration increased post-fatigue on both sides (10.3 ± 1.2 to 11.2 ± 1.3 ms on the affected side and 9.9 ± 1.7 to 11.2 ± 1.9 ms on the less-affected side, *p* = 0.001 and p = 0.02, respectively), and changes in action potential duration tended to be smaller in subjects with greater impairment (*p* = 0.04). This study presents evidence of both central and peripheral fatigue at the MU level during isometric fatiguing contraction for the first time in stroke survivors. Together, these preliminary observations indicate that the response to an isometric fatiguing contraction differs between the affected and less-affected side post-stroke, and may suggest that central mechanisms observed here as changes in firing rate are the dominant processes leading to task failure on the affected side.

## Introduction

Over the past decade, marked advancements in the acute management of stroke have led to an increase in the number of stroke survivors living with neurological disabilities (Feigin et al., 2014). One common limiting factor in the motor rehabilitation of stroke survivors is the prevalent loss of strength on the side of the body contralateral to the stroke lesion. This muscle weakness post-stroke has been attributed to alterations in the descending voluntary command, and to anatomical and physiological changes within the muscle (McComas et al., 1971; Bourbonnais and Noven, 1989; Dattola et al., 1993). Previous studies have identified impairments in voluntary muscle activation (Riley and Bilodeau, 2002; Knorr et al., 2011; Bowden et al., 2014; Hoffmann et al., 2016), altered motor unit (MU) firing rates (Rosenfalck and Andreassen, 1980; McNulty et al., 2014), a reduced ability to modulate MU firing (Gemperline et al., 1995; Mottram et al., 2014; Li et al., 2015) and abnormal MU recruitment patterns (Tang and Rymer, 1981; Hu et al., 2015, 2016), all of which may contribute to muscle weakness post-stroke.

In addition to enduring muscle weakness, stroke survivors may experience increased susceptibility to muscle fatigue. Both central and peripheral factors can contribute to the overall manifestation of fatigue, which can be defined as a transient exercise-induced reduction in the force-generating capacity of muscle (Bigland-Ritchie and Woods, 1984). The few studies that have investigated fatigue in stroke survivors during voluntary contractions have reported relatively higher central fatigue on the affected side when compared to the less-affected side and healthy controls (Riley and Bilodeau, 2002; Knorr et al., 2011). Central fatigue encompasses both decreases in descending motor commands to spinal motoneurons, and reduced excitatory afferent input, as well as decreases in motoneuron responsiveness due to changes in intrinsic properties or inhibitory afferent input (Gandevia, 2001). Conversely, in these studies stroke survivors showed lower levels of peripheral fatigue on the affected side. Peripheral fatigue refers to changes occurring beyond the motoneuron, including changes within the muscle fibers. Central fatigue was assessed in the stroke studies using twitch interpolation to quantify voluntary muscle activation (Riley and Bilodeau, 2002; Knorr et al., 2011), and peripheral fatigue was evaluated using the compression of the surface EMG power spectrum (Svantesson et al., 1999; Riley and Bilodeau, 2002) and changes in maximal twitch torque (Knorr et al., 2011).

Changes at the level of the single MU during muscle fatigue post-stroke have not yet been investigated. In the present study, we examine the hypothesis that there will be a greater loss in central activation during a sustained fatiguing contraction, and directly post-fatigue, on the affected side in hemispheric stroke. If higher central fatigue is present on the affected side, subjects may experience greater difficulty maintaining MU firing during a fatiguing contraction, as well as a diminished capacity to regulate MU firing rate directly post-fatigue. Changes in the excitability of the surface and tubular membranes of the muscle fiber on the affected side may also be lower post-fatigue (i.e., a marker of lower peripheral fatigue) which would manifest as smaller increases in the MU action potential duration (Andreassen and Arendt-Nielsen, 1987; Houtman et al., 2003; Fortune and Lowery, 2009).

In the present study, surface EMG decomposition was used to identify individual MU activities from non-invasive surface recordings. This provides direct information on both the MU discharge rates and the action potential duration, which is more closely correlated with muscle fiber conduction velocity and changes in muscle fiber excitability than indirect estimates from the surface EMG power spectrum. Samples of simultaneously active MUs were detected during short contractions (at 20% MVC) before and directly after a sustained isometric contraction of the first dorsal interosseous muscle in chronic stroke survivors. MUs were also detected during the sustained contraction (at 30% MVC).

The results of this study show that in chronic stroke survivors, MUs on the affected side displayed a greater decline in firing rate during sustained, fatiguing isometric contractions, than on the less-affected side. Furthermore, a greater change in MU firing rate was observed on the affected side immediately post-fatigue. Mean MU firing rates increased following the recovery period on the less-affected side but not on the affected side. A significant increase in action potential duration was observed on both sides post-fatigue. On the affected side, the magnitude of the change tended to be lower in subjects with greater impairments post-stroke. In combination, these results suggest that during sustained isometric fatiguing contractions in stroke survivors, central mechanisms play a greater role on the affected side, when compared to the less-affected side, and likely contribute to difficulties maintaining force reported post-stroke.

## Materials and Methods

### Experimental Procedure

**FIGURE 1:**
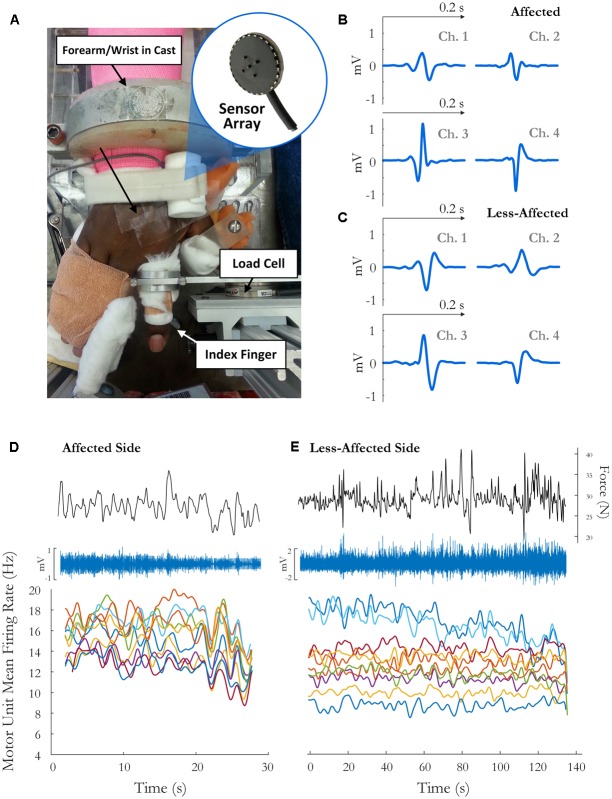
**(A)** Experimental setup and sample motor unit (MU) action potentials (spike-triggered average) on each channel for the **(B)** affected side and **(C)** the less-affected side. The force trace, surface EMG signal (on the highest amplitude channel), and time-varying MFR of MUs over the fatiguing contraction on the**(D)** affected and **(E)** less-affected side in a single subject (obtained by low-pass filtering the impulse train with 2 and 5 s Hanning windows, respectively).

Written informed consent was obtained for 12 stroke survivors (7 female, age 60 ± 7 years) to participate in this study, and the experimental protocols were approved by the Institutional Review Board at Northwestern University, **Table 1**. The force and EMG activity of the first dorsal interosseous muscle was examined during isometric abduction of the index finger, on both the contralateral and affected sides. The proximal phalanx of the index finger was fixed to a ring–mount interface attached to a load cell (ATI, Inc., 3226), and forces were recorded from the *x* (abduction/adduction) and *y* (extension/flexion) directions, **Figure 1A**. Surface EMG was recorded from the FDI using a surface sensor array (Delsys, Inc.) that consisted of five cylindrical probes (0.5 mm diameter) located at the corners and at the center of a 5 × 5 mm square (Nawab et al., 2010), and a reference electrode on the skin surface of olecranon. Pairwise differential recordings of the five electrodes yielded four channels of surface EMG signals, which were amplified and filtered between 20 Hz and 2 kHz). The signals were sampled at 20 kHz and stored on a computer for further processing.

**Table 1:** Clinical details on each stroke survivor, including time since stroke, location of brain lesion, the Upper-Extremity Fugl-Meyer Scale (FMUE) for the affected side and the MVC force ratio on the affected and less-affected sides.

Subject	Sex	Age	Years post-stroke	Type of stroke	Location	Fugl-Meyer Scale	MVC ratio (affected/less-affected side) (%)
1	F	61	8	Hemorrhagic	Cortical and subcortical	49	59
2	F	56	6	Ischemic	Cortical	51	75
3	M	69	11	Uncertain	Uncertain	18	31
4	M	50	5	Ischemic	Subcortical	54	91
5	M	58	3	Ischemic	Subcortical	53	68
6	F	70	7	Ischemic	Cortical and subcortical	52	23
7	M	59	11	Ischemic	Subcortical	52	67
8	F	62	9	Hemorrhagic	Cortical	53	92
9	M	48	7	Ischemic	Cortical and subcortical	60	90
10	F	55	14	Hemorrhagic	Cortical and subcortical	36	58
11	F	62	16	Hemorrhagic	Subcortical	19	27
12	F	69	15	Uncertain	Uncertain	28	75


*FMUE was assessed by a research physical therapist within 3-month period of the study. FMUE Scale scores <31 correspond with “no to poor” upper extremity capacity, 32–47 represent “limited capacity,” 48–52 represent “notable capacity,” and 53–66 represented “full” upper extremity capacity (Hoonhorst et al., 2015)*.

The experimental procedure was similar to that performed in healthy subjects, outlined in detail in McManus et al. (2015). Briefly, the maximal voluntary contraction (MVC) was determined as the highest force achieved during two or three short (3 s) maximum contractions, separated by a 1 min rest period, where the maximum force between trials lay within 10% of each other. Following the MVC, subjects performed a series of four isometric pre-fatigue voluntary contractions. The force trajectory for each contraction consisted of a 3-s quiescent period for baseline noise calculation, an up-ramp increasing at 10% MVC/s, a constant force of 20% MVC for 10 s, a down-ramp decreasing at 10% MVC/s, and a final 3 s quiescent period. After the four pre-fatigue trials, a sustained isometric contraction was performed at 30% MVC until task failure, defined as the point at which the subject’s force dropped 10% below the required output for 5 s or longer. Additional verbal encouragement was provided during the contraction to ensure that the force level was maintained for as long as possible. A single MVC was performed directly following task failure, and a series of four short duration contractions at 20% MVC, identical to those performed pre-fatigue, were performed post-fatigue with no rest period between trials to minimize recovery. Subjects were then allowed a 10-min recovery period before a series of four more trapezoidal trajectories at 20% MVC. For each condition, the goal was for the subject to perform four 20% MVC trials both pre- and post-fatigue. The number of trials completed, however, was higher on the affected side in some subjects in order to get the required number of trials with a steady force trace (4.8 ± 1 trials pre-fatigue and 4.4 ± 0.8 trials post-fatigue). The trial with the highest combined ranking, in terms of the steadiness of the force trace (low standard deviation) and the number of accepted MUs, was chosen to represent each condition.

### Data Analysis – Motor Unit Acceptance

Discriminable MUs were extracted from the surface EMG signal using the decomposition EMG system (Delsys, version 4.1.1.0). The decomposition algorithm is outlined in detail in Nawab et al. (2010) and De Luca and Hostage (2010). For each detected MU, the output of the decomposition algorithm consisted of the MU firing times and four motor unit action potential (MUAP) waveforms corresponding to four pairs of bipolar electrode channels.

The identified firing times for each MU were used to spike-triggered average (STA) the surface EMG signal on each channel, resulting in four representative STA MU action potential estimates for each MU. MUAP duration was estimated as the time between the zero crossing before the first positive peak of the action potential and the zero crossing after the last positive peak. The variation of the spike-triggered averaged MU action potential template over time was quantified using a moving average window. A spike-triggered averaged MU action potential template estimate was calculated based on the firing events in each window and the window was shifted along the length of the surface EMG signal. The reliability of each detected MU was then assessed by comparing the STA template estimates across all windows, using two tests outlined in Hu et al. (2013). The first measure of reliability was obtained by calculating the coefficient of variation (CV) for the peak-to-peak amplitude of the MUAP templates detected in each window. For the second measure, the maximum linear correlation coefficient (CC) was computed between the STA MU action potential template estimate and the decomposition-estimated templates.

Motor units were required to have an average CV in action potential amplitude <0.3 and CC (between the STA MUAP estimate and the decomposition MUAP template) >0.7 across all four channels to be selected for further analysis. In addition, MUs were required to have a CC > 0.8 and CV < 0.2 (0.25 for the longer fatiguing contraction) on at least one of the two channels with the highest MU action potential amplitude. A moving average window of 2 s length and 0.5 s time step was used to obtain the MUAP template for the short contractions pre- and post-fatigue and a window length of 4 s with a 1 s time step was used for the long fatiguing contraction. A minimum average of five MUs was required over the three trials (pre-fatigue, post-fatigue, and following the recovery period) for each subject. These units must also be recruited across a range of force levels, with a mean range of recruitment threshold defined as 5% MVC (with force normalized to subject MVC). MU data from 11 of the 12 subjects satisfied both criteria and was used in further analysis.

Motor unit mean firing rates (MFR) were analyzed during the sustained fatiguing contraction and during the short duration contractions before and after fatigue. Firing rate analysis was restricted to periods of relatively steady force production (standard deviation ≤ 3% MVC). Any overshoot during the initial increase to the required force level was excluded from the analysis and firing trains were truncated at the point where the mean force was below 10% of the desired force level for 5 consecutive seconds. The change in firing rate during the sustained fatiguing contraction was examined for each MU by fitting a least-squares regression line to the instantaneous firing rate data. For each accepted MU, the slope and intercept of the line was obtained, describing the initial MU firing rate and the change in MFR over the course of the fatiguing contraction.

### Data Analysis – Statistics

For each subject, the median MU MFR and action potential duration were obtained in the pre-fatigue, post-fatigue, and recovery trials. To focus on the within-subject effect of fatigue and minimize the contribution of inter-subject variance to the visual representation of results, the median from the pre-fatigue, post-fatigue, and post-recovery contractions for a given subject was normalized by subtracting that subject’s mean for the three contractions minus the grand mean of all subjects before generating the boxplot figures (Loftus and Masson, 1994).

A two-way within-subjects (or repeated measure) analysis of variance (ANOVA) was conducted to compare the change in each parameter across the pre-fatigue, post-fatigue, and recovery states, and on the affected and less-affected sides. Mauchly’s Test of Sphericity was implemented to check the assumption of sphericity, and if violated, a Greenhouse–Geisser correction was applied to the data. Pairwise differences between conditions were conducted using Fisher’s Least Significant Difference test with Bonferroni correction for the affected and less-affected sides. The changes in median MU firing rate and action potential duration from pre- to post-fatigue conditions, and from post-fatigue to recovery, were compared between the two sides with a two-sided paired *t*-test.

The change in MU firing rate during the sustained fatiguing contraction was examined using linear regression, and the *t*-statistic was used to test for a significant increase or decrease in motor firing rate. The relationship between initial MU firing rate (the intercept of the regression line) and the change in firing rate (slope of the line) was examined using a Pearson product-moment correlation. For each subject, the root-mean-squared (RMS) value of the EMG signal was calculated during the fatiguing contraction on the highest amplitude channel, using a 2 s time window with a 1 s time-step. The percentage change in RMS-EMG amplitude was then calculated by fitting a least-squares regression line to the RMS value of the EMG signal over time. The percentage change in the median frequency of the surface EMG power spectrum and in the CV of the force was obtained using the same window and time-step.

The relationships between Fugl-Meyer score and changes in median MU action potential duration, and between MU firing rates and action potential duration, were investigated with a Spearman’s rank-order correlation. An alpha level of 0.05 was used for all statistical tests, and the effect size is reported as omega squared (ω^2^) for the two-way ANOVA and as Hedges’ *G* (*g*) for the paired *t*-tests.

## Results

We first examined the properties of MUs detected on the more-affected and less-affected side in each stroke survivor. MU MFRs were compared between the two sides, in addition to differences in the action potential duration. We then investigated the effect of fatigue on MU firing rate and action potential duration by comparing values recorded during the short trials conducted pre- and post-fatigue. Changes in individual MU MFRs were also examined over the course of the sustained fatiguing contraction.

### Motor Unit Properties on the Affected and Less-Affected Sides Pre-fatigue

The average number of MUs detected pre-fatigue was 18.2 ± 7 and 22 ± 3.2 on the affected and less-affected sides. The corresponding averages post-fatigue were 16.8 ± 7 and 22.6 ± 3.8, respectively. Out of the total number of MUs detected, 59% of MUs were accepted for further analysis on the affected side and 52% were accepted on the less-affected side during the short contractions at 20% MVC. During the fatiguing contraction at 30% MVC, 48 and 47% of MUs were accepted on the affected and less-affected side, respectively.

Motor unit action potential duration was not significantly different between the affected and less-affected side when examined across all subjects (10.3 ± 1.2 vs. 9.9 ± 1.7 ms, *p* = 0.5). MU MFRs were similar on the affected and less-affected sides pre-fatigue (14 ± 4.4 and 13.7 ± 3.6 Hz, *p* = 0.70, respectively), though the firing rate CV was significantly higher on the affected side (0.09 ± 0.05 and 0.03 ± 0.01, respectively, *p* < 0.01, *g* = 1.4). A significant correlation was observed between the ratio of the MFR on each side and the ratio of the MUAP duration (*r* = 0.7, *p* = 0.01), with subjects for whom MUAP durations were longer on the affected side tending to also have higher MU MFRs on the affected side.

### MVC Force, MU Action Potential Duration, and Mean Firing Rate Pre- and Post-fatigue

**FIGURE 2:**
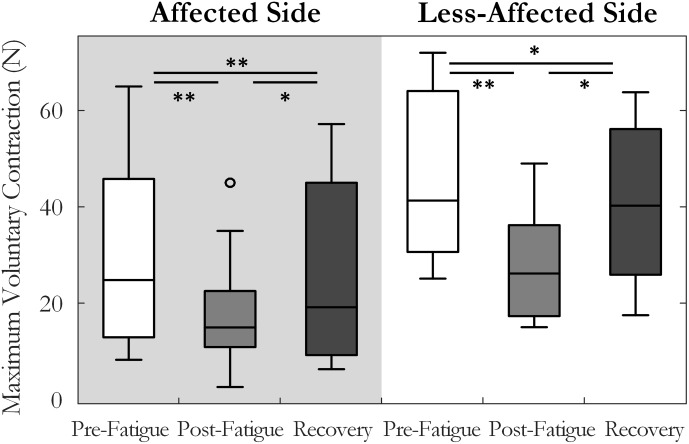
Median and interquartile range of the maximum voluntary contraction force across all subjects tested pre-fatigue, post-fatigue, and after the recovery period, on the affected and less-affected sides (^∗^*p* < 0.05, ^∗∗^*p* < 0.01).

A two-way repeated measures ANOVA was used to compare maximum voluntary index finger abduction force in the pre-fatigue, post-fatigue, and post-recovery states. The results indicated a significant change in MVC across the three states [*F*(1.3,14.6) = 21.8, *p* < 0.001, ω^2^ = 0.62], **Figure 2**. Lower MVC forces were recorded in each state on the affected side compared to the less-affected side [*F*(1,11) = 33.3, *p* < 0.001, ω^2^ = 0.7]. *Post hoc* tests revealed a significant decrease in maximum force post-fatigue on both sides (*p* < 0.01, both). The time to task failure varied greatly among subjects (143 ± 160 and 208 ± 73 s, on the affected and less-affected side, respectively) and was not significantly different between sides (*p* = 0.07). After 10 min of rest, the MVC force increased but remained significantly lower than pre-fatigue values on both sides (*p* = 0.036 on the affected and *p* = 0.01 on the less-affected side), **Figure 2**.

**FIGURE 3:**
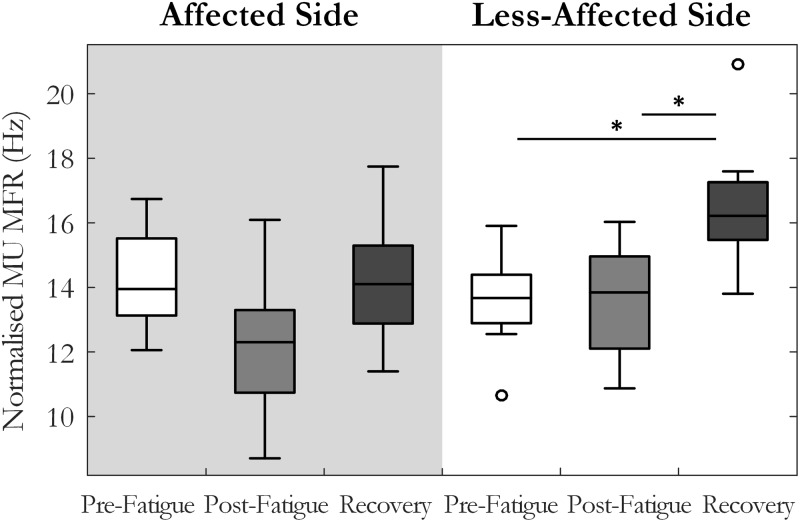
Median and interquartile range of median MU firing rate across all subjects from MUs detected pre-fatigue, post-fatigue, and after the recovery period, on both the affected and less-affected sides (^∗^*p* < 0.05).

Motor unit MFRs changed significantly across the pre-fatigue, post-fatigue, and recovery states [*F*(2, 20) = 4.7, *p* = 0.02, ω^2^ = 0.24], **Figure 3**. There was also a significant interaction between state and side (affected or less-affected) for MU firing rate [*F*(2,20) = 4.37, *p* < 0.05, ω^2^ = 0.23], **Figure 3**. *Post hoc* tests revealed that MFRs were significantly higher following the 10-min recovery period than those reported pre-fatigue on the less-affected side (13.7 ± 3.6 and 16.4 ± 5.6 Hz, *p* = 0.03, pre-fatigue and post-recovery, respectively).

**FIGURE 4:**
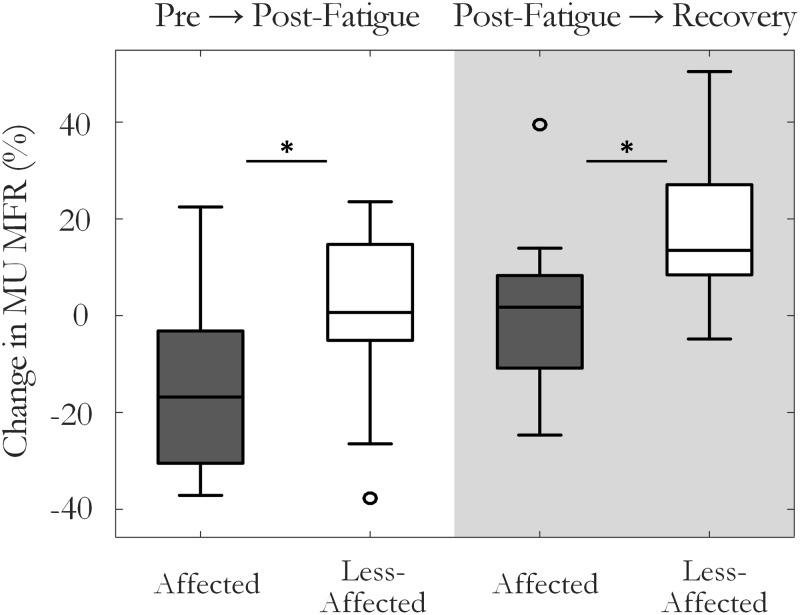
Median and interquartile range of the percentage change in MU firing rate on the affected and less-affected sides, comparing MUs detected pre-fatigue and post-fatigue, and pre-fatigue with MUs detected after the recovery period (^∗^*p* < 0.05).

The percentage change in MU MFR from the pre-fatigue to post-fatigue trials was calculated to compare the response to the sustained fatiguing contraction on the affected and the less-affected sides, **Figure 4**. The affected side exhibited a reduction in MU MFRs post-fatigue that was not observed on the less-affected side (-13.5 ± 20% on the affected and 0.1 ± 19% on the less-affected side, respectively, *p* = 0.04, *g* = -0.67). The percentage change in MU firing rate from pre-fatigue to post-recovery trials also differed between the affected (0.5 ± 20%) and less-affected sides (19.3 ± 17%, *p* = 0.03, *g* = -1.1), with higher MU firing rates observed on less-affected side following the recovery period.

**FIGURE 5:**
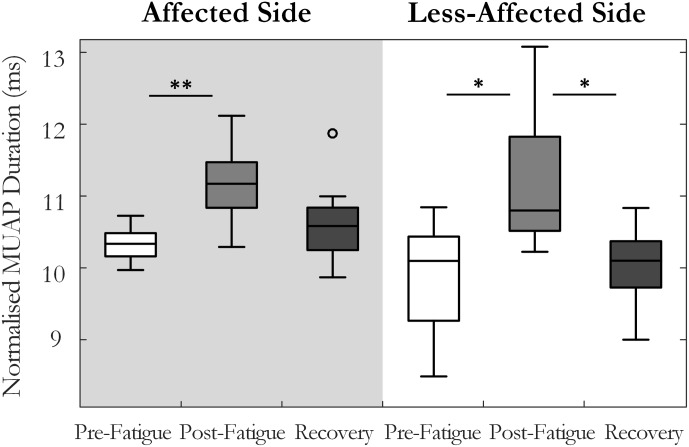
Median and interquartile range of the median MU action potential duration across all subjects from MUs detected pre-fatigue, post-fatigue, and after the recovery period, on both the affected and less-affected sides (^∗^*p* < 0.05, ^∗∗^*p* < 0.01).

**FIGURE 6:**
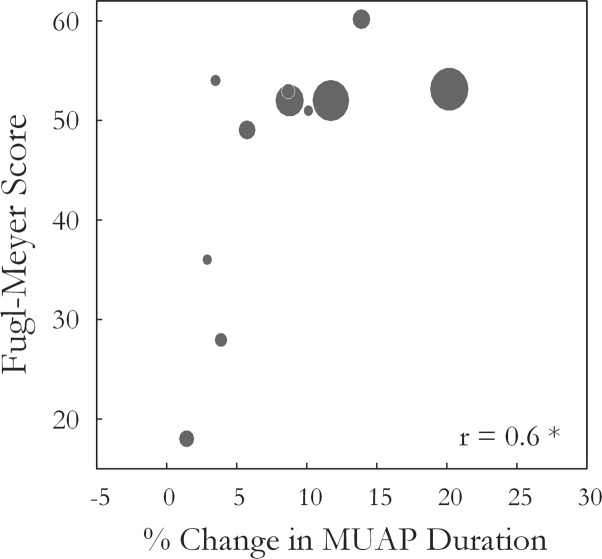
The Wrist and Hand Upper Extremity Fugl-Meyer score for each subject is plotted against the percentage change in median MUAP duration from pre- to post-fatigue observed for that subject (*r* = 0.6, *p* = 0.04). Larger circles indicate longer times to task failure for the sustained fatiguing contraction.

Motor unit action potential duration also changed significantly over the three states [*F*(1.34,13.4) = 10.35, *p* < 0.01, ω^2^ = 0.44]. An increase in MU action potential duration was observed post-fatigue on both the affected (10.3 ± 1.2 to 11.2 ± 1.3 ms, *p* = 0.001) and less-affected sides (9.9 ± 1.7 to 11.2 ± 1.9 ms, *p* = 0.02), **Figure 5**. Following the recovery period, MUAP duration recovered and did not differ significantly from pre-fatigue values (10.6 ± 1.2 ms, *p* = 0.2, on the affected side and 10.1 ± 1.2 ms, *p* = 0.5, on the less-affected side). Subjects that were more impaired post-stroke, as evidenced by their Upper Extremity Fugl-Meyer scores, showed smaller changes in MU action potential duration following the fatiguing contraction (*r* = 0.6, *p* = 0.04), **Figure 6**. The percentage change in MUAP duration from pre- to post-fatigue did not differ significantly between the affected (8.2 ± 6%) and less-affected sides (14.2 ± 17%, *p* = 0.19), though there was a greater range of changes in MUAP duration on the less-affected side, Supplementary Figure S2.

### Motor Unit Firing Rate, Surface EMG, and Force during the Sustained Fatiguing Contraction

**FIGURE 7:**
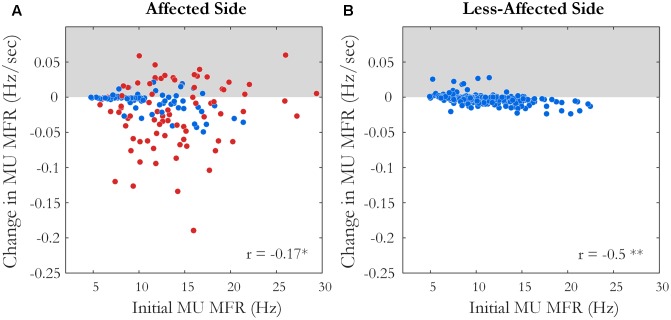
The slope and intercept of the linear fit to the change in MU MFR over the course of the sustained fatiguing contraction over all subjects on **(A)** the affected side, ^∗^*p* < 0.05 and **(B)** the less-affected side, ^∗∗^*p* < 0.01. Each data point represents an accepted MU; red points indicate MUs from the affected side of subjects with an endurance time for the fatigue task <75% of the shortest time to task failure on the less-affected side.

Motor unit firing times were obtained from the decomposed surface EMG signal during the fatiguing contraction at 30% MVC in 10 of 12 subjects, with data from an exemplar subject shown in **Figure 1**. A similar percentage of MUs exhibited a statistically significant decrease in MFR over time on the affected and less-affected sides (28 and 30%, respectively). Only a small number of units exhibited a significant increase in firing rate (4 and 6%, respectively). MU MFRs decayed faster on the affected side during the fatiguing contraction than on the less-affected side over all subjects (-0.02 ± 0.03 and -0.004 ± 0.003 Hz/s, respectively, *p* = 0.045, *g* = -0.94), **Figure 7**. However, there was no significant difference in the absolute decrease in MU firing rate (-0.85 ± 0.8 Hz on the affected side and -0.48 ± 0.36 Hz on the less-affected side, *p* = 0.17). When MUs were pooled over all subjects, lower threshold MUs with higher MFRs tended to show greater absolute decreases in firing rate during the fatiguing contraction on the affected side (*r* = -0.18, *p* = 0.02) and less-affected side (*r* = -0.5, *p* < 0.001), **Figure 7**.

The variability of the force (CV) during the fatiguing contraction was higher on the affected side than on the less-affected side (0.1 ± 0.07 and 0.04 ± 0.02, *p* < 0.001, respectively), and increased on both sides as the contraction progressed (108 ± 121% on the affected side and 40 ± 72% on the less-affected side, with no significant difference between sides, *p* = 0.3). Force variability was also higher on the affected side during the short contractions pre-fatigue (0.07 ± 0.05 and 0.03 ± 0.01, *p* < 0.05), and there was no significant change in force variability post-fatigue on either the affected or less-affected side (*p* = 0.4 and *p* = 0.7, respectively). The RMS-EMG amplitude during the first quarter of the fatiguing contraction was lower on the affected side when compared to the less-affected side (0.13 ± 0.1 and 0.28 ± 0.2 mV, *p* < 0.01), with no clear change as the contraction progressed on either side (-1.6 ± 69 and -13 ± 44%, *p* = 0.6, respectively). There was no difference in the median frequency of the surface EMG on the affected (146 ± 35 Hz) and less-affected sides (174 ± 61 Hz, *p* = 0.3) during the first quarter of the fatiguing contraction. However, there was a greater decrease in median frequency during the contraction on the less-affected side (-44 ± 15%) when compared to the affected side (-16 ± 19%, *p* < 0.01).

### Motor Unit Properties in Subjects with Matched Force Levels

As the majority of subjects had a large difference in MVC between the affected and less-affected side, there is the additional confounding factor of different absolute force levels when comparing changes in MU properties. However, in five subjects, the fatiguing contraction was performed at similar absolute forces on the affected and less-affected sides (<25% difference in MVC). In this subset, two subjects were unable to sustain the fatiguing contraction on the affected side for more than 25% of the time obtained on the less-affected side. These subjects had shorter MU action potential durations and lower MU MFRs on the affected side compared to the less-affected side. This could indicate recruitment of a greater proportion of the MU population, including higher threshold MUs with higher muscle fiber conduction velocities and shorter duration action potentials to achieve the target force on the affected side. The lack of reserve MUs available for recruitment during the fatiguing contraction may have resulted in the early task failure. These subjects exhibited small changes (<10%) in MU action potential duration on the affected side post-fatigue, alongside a reduction in MU MFR (-17%, -10%). Of the remaining three subjects, two showed significant changes in MU action potential duration on the less-affected side (>25%), with smaller changes observed on the affected side (>15%) and the third subject showed little change in MU action potential duration on the affected and less-affected side (<5%). When changes in MU MFR during the fatiguing contraction were examined, all subjects with matched force levels exhibited a significantly larger rate of decrease in MU MFR on the affected side when compared to the less-affected side (-0.04 ± 0.02 and -0.002 ± 0.003 Hz/s, respectively).

## Discussion

In this study, changes in MU properties were investigated prior to, during, and directly after a submaximal, isometric fatiguing contraction in chronic stroke survivors. The ability to investigate adaptations in MU firing rate of many MUs using surface EMG decomposition gives a unique insight into the regulation of MU behavior and how this contributes to the overall manifestation of fatigue in stroke survivors. During the sustained fatiguing contraction, MUs on the affected side displayed a greater decline in firing rate than those on the less-affected side. Furthermore, a greater change in MU firing rate was observed on the affected side immediately post-fatigue. Mean MU firing rates increased following the recovery period on the less-affected side but not on the affected side. Changes in MUAP duration post-fatigue tended to be smaller on the affected side in subjects with greater impairment, indicating lower levels of induced peripheral fatigue. These observations suggest that central fatigue was more dominant on the affected side when compared to the less-affected side, resulting in greater difficulty maintaining or augmenting MU firing rates during and directly post-fatigue.

### Comparison of MU Properties on the Affected and Less-Affected Sides Pre-fatigue

No significant difference was observed in either action potential duration or MFR between sides pre-fatigue. Subjects with relatively longer MU action potential duration on the affected side tended to have higher MU MFRs on the affected side compared to the less-affected sides. Previous studies have reported slower muscle fiber conduction velocities in certain, but not all muscles (Yao et al., 2015; Conrad et al., 2017), and longer MUAP durations on the affected side post-stroke using intramuscular EMG (Lukács, 2005). Lower firing rates on the affected side have been reported when comparing firing rates recorded at the same absolute force on both sides, using intramuscular (Gemperline et al., 1995; Chou et al., 2013) and surface EMG decomposition techniques (Suresh et al., 2011; Hu et al., 2012; Li et al., 2015). A tendency toward lower MU MFRs has also been observed on the affected side at the same relative force levels, similar to what was compared here, using intramuscular EMG (Rosenfalck and Andreassen, 1980; Hu et al., 2006) and surface EMG during low level contractions (McNulty et al., 2014).

### Motor Unit Firing Rate and Action Potential Characteristics during the Fatiguing Contraction and Directly Post-fatigue

During the fatiguing contraction, there was a significant decline in MU MFR in approximately 30% of all accepted MUs, on both the affected and less-affected side, **Figure 7**. The average magnitude of this decline was greater on the affected side, even in subjects that performed the fatiguing contraction at similar force levels on both sides. The mean time to task failure was lower on the affected side, though did not reach statistical significance due to the large variability across subjects. This is similar to the findings of previous studies that have used forces at the same percentage of maximal effort on each side to examine fatigue in various muscles post-stroke (Sunnerhagen et al., 1999; Svantesson et al., 1999; Riley and Bilodeau, 2002; Hyngstrom et al., 2012).

The change in MU firing rate from pre- to post-fatigue contractions was also significantly larger on the affected side than the less-affected side, with lower MU firing rates observed post-fatigue on the affected side, **Figure 4**. Failure to sustain steady MU discharge likely contributed to an inability to maintain force output on the affected side during the fatiguing contraction, particularly as recruitment often occurs over a compressed force range post-stroke and there may be few MUs available to recruit (Tang and Rymer, 1981; Hu et al., 2015). This decline in MU MFR may be mediated by the partial loss of excitatory efferent drive from the descending motor pathways to the segmental motoneurons and interneurons following stroke (McComas et al., 1973; Dattola et al., 1993; Lindberg et al., 2007), though impairments in descending corticospinal connections (Bowden et al., 2014), motor axons (Jankelowitz et al., 2007), and changes in intrinsic motoneuron properties could also play a role. Larger reductions in MU MFR post-fatigue were also associated with poorer recovery of muscle force capacity on the affected side following the rest period, Supplementary Figure 1C. On the affected side, the change in RMS-EMG amplitude was correlated with the change in MU MFR during the fatiguing contraction, with little evidence of MU recruitment, Supplementary Figure 1A. These relationships were not found on the less-affected side, Supplementary Figures 1B,D, respectively. Collectively, these observations provide evidence that changes in central mechanisms are the dominant processes contributing to fatigue on the affected side. This aligns with the findings of previous studies that have reported a greater reduction in voluntary muscle activation on the affected side during sustained submaximal and maximal contractions using twitch interpolation techniques (Riley and Bilodeau, 2002; Knorr et al., 2011).

Smaller changes in MU action potential duration post-fatigue were observed on the affected side in subjects with greater impairment, **Figure 6**. Consistent with this, the median frequency decreased by less during the fatiguing contraction on the affected side. Better muscle perfusion and lower intramuscular pressure at lower target forces on the affected side may have reduced metabolic accumulation (Hunter, 2009), which would present as smaller changes in MUAP duration. However, subjects who performed the fatiguing contraction at similar forces with comparable times to task failure on both sides also exhibited smaller changes in MUAP duration on the affected side (>15%) than on the less-affected side (>25%). There may also be a reduction in the proportion of the MU pool capable of being recruited with the atrophy and/or functional loss of larger, high-threshold MUs (Edström, 1970; Dattola et al., 1993; Lukács et al., 2008; Klein et al., 2013). A final contributing factor may be the length of the fatiguing contraction, as subjects that held the contraction for a very short amount of time on the affected side exhibited little change in MUAP duration. Thus, lower peripheral fatigue on the affected side in the present study may arise as a result of lower absolute force levels, the early cessation of the fatigue task due to higher central fatigue, recruitment of a greater proportion of fatigue-resistant Type I fibers within the paretic muscle, or a combination of these factors. Lower levels of peripheral fatigue on the affected side have been reported in previous studies investigating peripheral fatigue using indices derived from surface EMG during voluntary contractions in stroke survivors (Svantesson et al., 1999; Riley and Bilodeau, 2002).

The increase in MUAP duration reported on both the affected (8 ± 6%) and less-affected side (14 ± 17%) was considerably lower than that observed post-fatigue in young, healthy subjects using a similar protocol (25 ± 14%) (McManus et al., 2015). Subject age is likely to have contributed to this discrepancy, as a characteristic shift in muscle fiber-type distribution toward Type I fibers occurs with aging, with a denervation of fast-twitch fatigable fibers and a subsequent reinnervation of adjacent slow-twitch fatigue resistant fibers.

In young subjects, lower MU MFRs were consistently observed post-fatigue. However, on the less-affected side, more than half the stroke survivors exhibited MU MFRs that were higher or unchanged post-fatigue. The reduction in MU firing rates and recruitment thresholds that accompanies the shift toward Type I fibers and slowing contractile properties with age (Erim et al., 1999) could account for the different response to fatigue on the less-affected side in stroke survivors. In addition, the ability to voluntarily activate the muscle may be impaired on the less-affected side in some subjects (Bowden et al., 2014). Subjects that were able to increase MU MFRs post-fatigue on the less-affected side were more likely to exhibit greater changes in MUAP duration, suggesting the degree of peripheral fatigue experienced is related to the ability to voluntarily activate the muscle to its full capacity, Supplementary Figure 2C. The repeated short contractions pre-fatigue and long fatiguing contraction may have also increased MU MFR post-fatigue in some subjects, as repetitive muscle activation has also been shown to elicit improvements in motor performance and an increase in EMG in both stroke survivors and healthy subjects (Massie et al., 2016).

### Limitations

The surface EMG decomposition method used in this study was chosen because its algorithm makes no assumptions about the characteristics of the MU action potential waveforms or the statistics of the MU firing instances (Kline and De Luca, 2014), both of which may be altered post-stroke. However, the decomposition method comes with certain caveats; the accuracy of the decomposition system for a particular MU can be influenced by the stability and the signal-to-noise ratio of its action potential waveform (Hu et al., 2014). In addition, the influence of MU synchronization on the algorithm’s accuracy has not been quantitatively assessed. Smaller MUs, more instability in the MU action potential waveform or higher levels of broad band MU synchronization post-stroke could make the algorithms more susceptible to firing time inaccuracies during the decomposition of surface EMG signals in stroke survivors, when compared to healthy individuals. To minimize the contribution of falsely identified firing instances, the stability of each MU action potential waveform was assessed to select the most reliable MU firing trains for further analysis (Hu et al., 2013). However, the contribution of firing instances missed by the decomposition system cannot be quantified and should be noted as a possible factor influencing MU MFRs in this study.

In the present study, the less-affected side was used as a control against the changes observed in MU firing rate and action potential duration on the affected side in stroke survivors. However, alterations in MU behavior are also observed on the less-affected side post-stroke, with higher MFRs reported when compared to healthy subjects (Hu et al., 2006; McNulty et al., 2014). Changes in MU contractile properties are also bilateral, and prolonged MU twitch contraction times (McComas et al., 1973; Young and Mayer, 1982; Frontera and Larsson, 1997) and longer muscle half relaxation times (Horstman et al., 2010) observed on both sides in stroke survivors when compared to data from age-matched controls. Thus, the response to fatigue on the less-affected side may differ from that of older, healthy controls due to bilateral changes occurring post-stroke. A final limitation of the study was the small sample size and low statistical power, which results in a reduced chance of detecting a true effect and also decreases the likelihood that a statistically significant result reflects a true effect (Button et al., 2013). Thus, a study with a larger sample size is needed to confirm these preliminary results.

## Conclusion

For the first time in stroke survivors, this study presents manifestations of both central and peripheral fatigue by examining the activity of a large number of simultaneously active MUs. Mean MU MFRs decreased more rapidly during the sustained contraction on the affected side when compared to the less-affected side. The change in MU MFRs from pre- to post-fatigue trials was also greater on the affected side. Although the change in action potential duration was not significantly different between sides, changes in MU action potential duration tended to be smaller in subjects with greater impairment. These results suggest that central mechanisms are the dominant processes during fatigue on the affected side. The present study is the first to describe the specific changes in MU firing rates and action potential duration during a sustained fatiguing contraction to the endurance limit in chronic stroke survivors. These measures provide indices to assess the prevalence of central and peripheral fatigue from surface EMG recordings in stroke survivors. This opens-up the possibility of exploring fatigue in other pathological disorders using a non-invasive, stimulation-free protocol. These alterations in MU behavior provide insight into strategies employed by stroke survivors to compensate for impaired muscle force.

## Ethics Statement

Written informed consent was obtained for each subject to participate in this study, and the experimental protocols were approved by the Institutional Review Board at Northwestern University.

## Author Contributions

LM, ML, and NS performed the conception and design of research. LM, XH, and NS performed experiments. LM, ML, and NS analyzed the data. LM, ML, WR, and NS interpreted the results of experiments. LM prepared figures. LM and ML drafted the manuscript. LM, ML, NS, and WR edited and revised the manuscript. LM, ML, WR, XH, and NS approved final version of the manuscript.

## Conflict of Interest Statement

The authors declare that the research was conducted in the absence of any commercial or financial relationships that could be construed as a potential conflict of interest.
