# Supplementary material for: Motor Unit Activity during Fatiguing Isometric Muscle Contraction in Hemispheric Stroke Survivors
Source: Front Hum Neurosci. 2017 Nov 24;11:569. doi: 10.3389/fnhum.2017.00569 (PMC5705653; doi:10.3389/fnhum.2017.00569)
Supplement: Supplementary file 1 [file Image_1.PDF]

Supplementary Material

Motor unit activity during fatiguing isometric muscle contraction in hemispheric stroke survivors

Lara McManus[1*], Xiaogang Hu[2], William Z. Rymer[3,4], Nina L. Suresh[3,4] and Madeleine M. Lowery[1]

[1]Neuromuscular Systems Lab, School of Electrical and Electronic Engineering, University College Dublin, Ireland

[2]Joint Department of Biomedical Engineering, University of North Carolina-Chapel Hill and North Carolina State University, NC 27599, USA

[3]Shirley Ryan AbilityLab, Chicago, IL 60611, USA

[4]Northwestern University, Evanston, IL 60208, USA

*** Correspondence:**
Lara McManus, lara.mcmanus@ucd.ie

1.1 Supplementary Figures

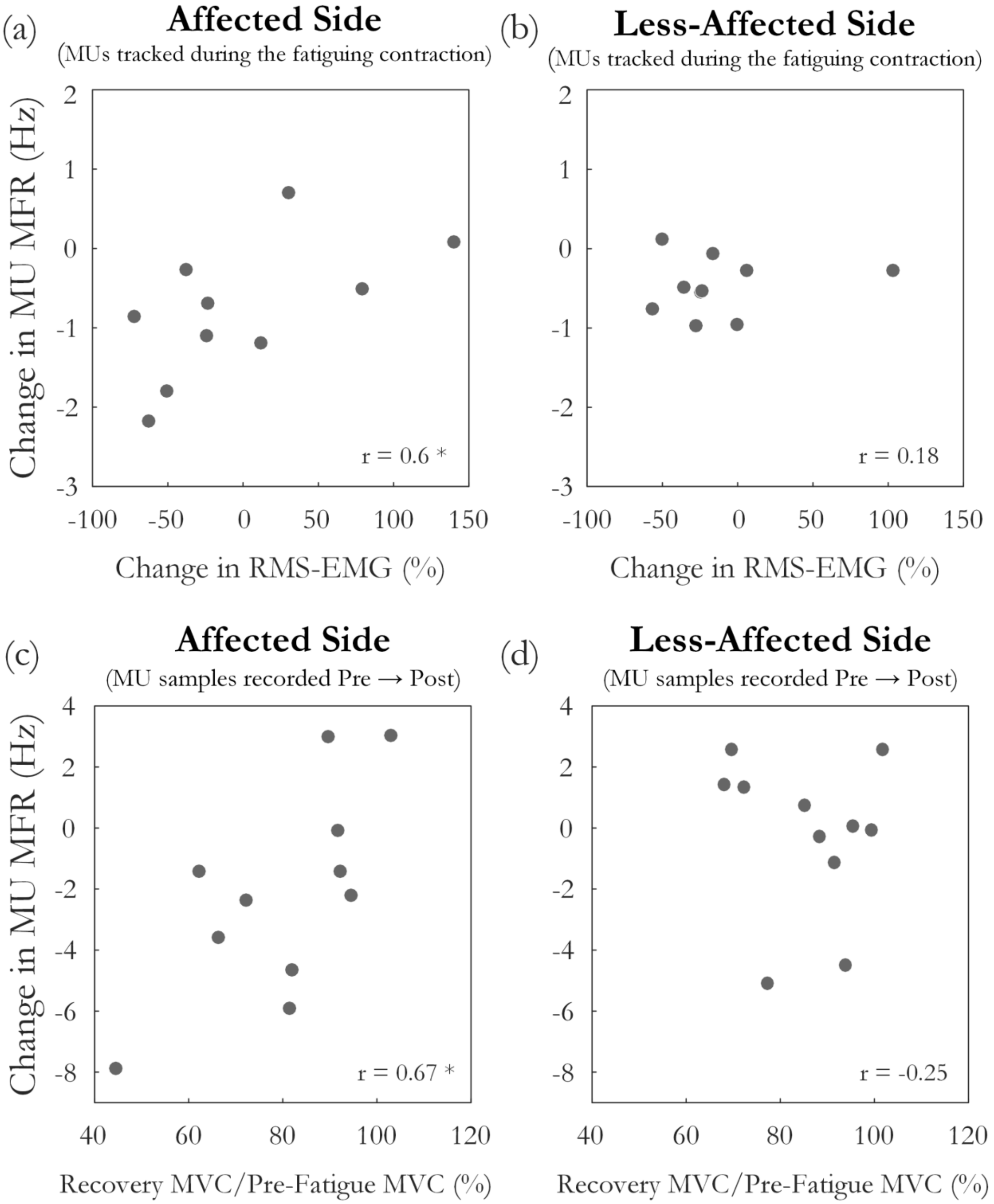

Supplementary Figure 1. The absolute change in mean firing rate in the same motor unit sample during the fatiguing contraction is plotted against the percentage change in RMS-EMG on (a) the affected side ($r = 0.6$, * $p = .05$) and (b) the less-affected side ($r = 0.18$, $p = .6$) for each subject. In three subjects, a large increase in RMS-EMG amplitude (> 70%) was observed, and the RMS-EMG

amplitude in these subjects appeared to be dominated by the amplitude of a small number of units. These subjects tended to have the lowest EMG amplitudes with the highest kurtosis values (a measure of signal sparsity), suggesting the increase was driven by the activity of a select number of units. The absolute change in mean firing rate in the separate motor unit samples recorded during the short contractions pre- and postfatigue against the percentage recovery of the subject MVC following the 10-minute rest period on the (c) affected side (r = 0.67, * p < .05) and (d) less-affected side (r = -0.25, p = .5).

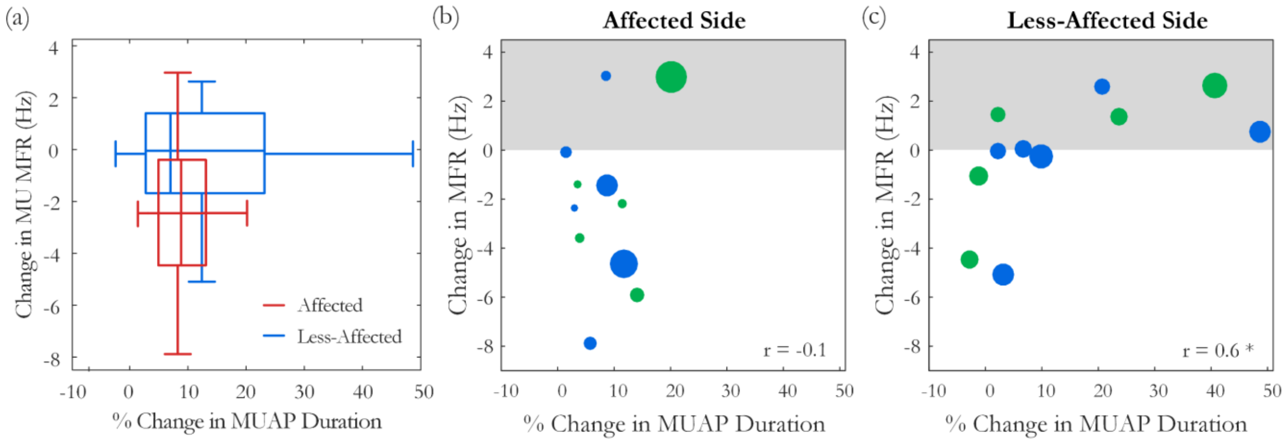

Supplementary Figure 2. (a) Two-dimensional boxplot of the absolute change in MU mean firing rate for each subject against the percentage change in median MUAP duration from pre- to postfatigue observed for that subject. The boxes represent the affected (red) and less-affected sides (blue) and summarizes the two distributions. In each direction, the box is limited by first and third quartile of the distributions. The middle line (horizontal or vertical) inside of the boxes indicates the median value. The same data is plotted for each subject on the (b) affected (r = -0.1, p = .7) and (c) less-affected sides (r = 0.6, * p < .05). Larger circles indicate longer times to task failure for the sustained fatiguing contraction, the circles in green show subjects with similar MVCs on the affected and less-affected sides (~75%).
